# Five‐year corneal cross‐linking outcomes: A Save Sight Keratoconus Registry Study

**DOI:** 10.1111/ceo.14177

**Published:** 2022-10-24

**Authors:** Alex C. Ferdi, Himal Kandel, Vuong Nguyen, Jeremy Tan, Francisco Arnalich‐Montiel, Marco Abbondanza, Stephanie L. Watson

**Affiliations:** ^1^ Specialty of Clinical Ophthalmology and Eye Health, Sydney Medical School Save Sight Institute, The University of Sydney Sydney New South Wales Australia; ^2^ Cornea & External Eye Diseases Hospital Universitario Ramón Cajal Madrid Spain; ^3^ Abbondanza Eye Centers Rome and Milan Italy

**Keywords:** adverse events, cross‐linking, keratoconus, outcomes

## Abstract

**Background:**

We aimed to determine the long‐term outcomes of epithelium‐off cross‐linking (CXL) in keratoconus patients.

**Methods:**

An observational registry study from 41 centres across 5 countries was carried out. Primary outcomes included the mean change in visual acuity (VA), Kmax, K2, and thinnest corneal thickness (TCT) at 1–5 years. Secondary outcomes included the percentage of eyes with worsening, stable and improving outcomes.

**Results:**

There were 976 eyes of 794 patients with 1‐year of complete follow‐up, 501 eyes with 2‐years, 355 with 3‐years, 235 with 4‐years and 162 with 5‐years. There was a significant improvement in mean VA from baseline by 3.7 logMAR letters (*p* < 0.001) in year 1, and 6.9 (*p* < 0.001) in year 5. Mean Kmax decreased by 1.2 dioptres (D; *p* < 0.01) in year 1. During subsequent years the Kmax flattening appeared sustained but this was not statistically significant. K2 flattened significantly from baseline in year 1 and then remained stable. At 1 year, 4.1% patients were poor responders to CXL in terms of VA, losing ≥15 letters. The proportion of the poor responders remained unchanged: 4.9% at 5‐years. The proportion of poor responders in terms of Kmax remained similar: 5.9% steepening by ≥2D at 1‐year and 7.5% at 5‐years. The proportion of K2 poor responders remained stable with 4.7% steepening by ≥2D at 1‐year and 5.8% at 5‐years.

**Conclusions:**

Cross‐linking is effective at stabilising keratoconus up to 5 years in most patients. However, a small proportion of eyes failed to stabilise and had reduced vision.

## 1 INTRODUCTION

Keratoconus is typically a bilateral progressive chronic corneal disorder leading to poor quality vision and reduced quality of life.^1^, ^2^, ^3^ Corneal cross‐linking (CXL) has shown great potential as a therapy for halting progression in keratoconus, with the Global Consensus on Ectasia describing it as an ‘extremely important’ innovation.^4^ Though initial reports of the procedure were encouraging, the procedure is not without risks^5^ and its outcomes can be variable. Clinicians require knowledge of the risks and benefits of a procedure to inform their clinical practice and patients.

Data on the outcomes from CXL are available from case reports, case studies and a few randomised controlled trials (RCTs). The existing studies, particularly the long‐term studies, are limited by the low sample size.^6^, ^7^, ^8^ RCTs have shown promising results in stabilising progressive keratoconus.^9^, ^10^, ^11^, ^12^ However, reporting of the number of patients exhibiting a good and poor response after CXL has been limited.^13^, ^14^ In addition, RCTs involve highly selective populations with important patient groups, such as those with comorbidities and pregnant women, excluded. The results of RCTs may not reflect routine clinical practice outcomes and maybe less powered to detect rare adverse events which require a large study sample size to capture.^15^ Adverse events from CXL range from the seemingly benign and typically reversible, including clinically significant corneal haze,^16^ to the more severe with the potential for significant permanent visual loss such as microbial keratitis, herpetic infection, and corneal melt or scarring.^5^, ^17^, ^18^, ^19^

It is essential to verify that the results from RCTs translate into routine clinical practice, however, these data on CXL outcomes are currently lacking. Of the studies in routine clinical practice that have been reported, few are prospective, have a long patient follow‐up, or are large multi‐centre studies.^20^, ^21^, ^22^ The Save Sight Keratoconus Registry is a prospectively designed registry collecting real‐world outcomes data on keratoconus patients.^23^, ^24^ It tracks the patient's treatment journey and provides benchmark reports for clinicians aiming to improve the practice of CXL.

We report CXL outcomes from patients in the prospectively designed Save Sight Keratoconus Registry (SSKR) from 32 centres across Australia, New Zealand, Spain and Italy. Visual acuity (VA), Kmax, K2, and thinnest corneal thickness (TCT) as well as adverse events were reported across 5 years of follow‐up.

## 2 METHODS

### 2.1 Design and setting

We analysed data from routine clinical practice in an observational study. Data was captured through the Fight Corneal Blindness! Projects' Save Sight Keratoconus Registry, which is based on the Fight Retinal Blindness! Project (https://savesightregistries.org).^25^ The registry is a web‐based internationally growing patient database for tracking natural history and treatment outcomes in keratoconus.^26^, ^27^ The clinicians using the SSKR in their routine clinical practice recorded patient demographics, ocular and systemic history, equipment, monitoring parameters (e.g., visual acuity, keratometry, pachymetry, and adverse events), and details of treatments including CXL.^26^, ^27^ At the baseline visit, demographic information including birth year, gender and ethnicity were recorded in addition to clinical information including prior CXL, refractive procedures including intra‐corneal ring segments (ICRS) or corneal grafts and relevant medical history. CXL treatment protocol features were recorded in the SSKR and were at the discretion of the treating clinician. Only epithelium‐off CXL procedures were included in this study to align with the most common treatment practice. Visual acuity was measured as “habitual visual acuity”^27^ in logMAR letters in the patient's usual visual correction method which was recorded as unaided, spectacles, or contact lenses. Visits in which a different refractive correction method was used to record VA than the pre‐CXL visit were excluded to prevent this confounding VA change results. For example, follow‐up visits with VA measured unaided when the pre‐CXL visit was measured with spectacles, were excluded. Oculus Pentacam was the tomographer used in a majority (69.5%) of cases.

Adverse events collected in the SSKR included clinically significant haze, corneal melt, corneal scarring, stromal oedema microbial keratitis, persistent epithelial defect, progressive keratoconus, recurrent erosion, sterile infiltrates, herpes simplex keratitis, herpes zoster keratitis, infectious endophthalmitis, macula oedema, non‐infectious endophthalmitis, and an intraocular pressure increase in response to steroids (“steroid response”). Corneal haze was only recorded as an adverse event if it was “clinically significant haze,” that is haze affecting vision as judged by the reviewing clinician.^28^ Treatment regimes including review frequency, topographer used, and contact lens removal protocol were carried out according to the routine practice of the clinician.

Local area Health Research Ethics Committees provided institutional ethics approval to public hospitals. Private practices received ethics approval from The Royal Australian and New Zealand College of Ophthalmologists' Ethics Committee. Informed consent was obtained from each participant and the tenets of Declaration of Helsinki were followed.

### 2.2 Study population

Study inclusion criteria included keratoconus patients who had undergone epithelium‐off CXL and who had not received any prior surgical intervention, including CXL, refractive procedures or ICRS. Patients enrolled in the registry before February 2021 completing 1–5 years of follow‐up after CXL were identified from 41 centres across five countries: Australia, New Zealand, Italy, France, and Spain. When patients had a procedure, only data leading up to these procedures were used.

### 2.3 Study outcomes

Primary outcomes included the mean change in visual acuity (VA), Kmax, K2, and thinnest corneal thickness (TCT) at 1–5‐years inclusively.

Secondary outcomes included the percentage of 1 to 5‐year completers with worsening, stable and improving VA, Kmax, K2, and TCT. Further outcomes included the CXL procedure protocols and the frequency of adverse events.

### 2.4 Statistical analysis

Descriptive statistics included mean, median, percentages, standard deviation (SD) and 95% confidence intervals (CIs) where appropriate. Changes in outcomes between the pre‐CXL visit and annual follow‐up visits were analysed using paired t‐tests. *p*‐Values from multiple comparisons were adjusted for using the Holm‐Bonferroni correction method.^29^ The analyses were conducted using the R software (Version 4.0.2; The R Foundation for Statistical Computing, Vienna, Austria).^30^

## 3 RESULTS

### 3.1 Study participants

There were 976 eyes of 794 patients, 68% of whom were male, with 1 year of complete follow‐up. Thirteen eyes underwent a second CXL procedure and no eyes underwent more than two procedures. At baseline, the mean age was 26.3 years (10.3 SD), VA 63.4 logMAR letters (roughly equivalent to 6/15 or 20/50 Snellen) (20.0 SD), Kmax 56.6D (8.2 SD), K2 50.3D (5.9 SD), and TCT 459 μm (43 SD). There were 501 eyes with 2‐years follow‐up, 355 with 3‐years, 235 with 4‐years and 162 with 5‐years. The demographics of the completers of each successive year of follow‐up are detailed in Table 1.

**TABLE 1:** Baseline characteristics of eyes undergoing cross‐linking with 1–5 years follow‐up

	1 year completers	2 year completers	3 year completers	4 year completers	5 year completers
Eyes	975	517	367	245	168
Patients	791	434	301	194	131
Baseline age	26.4 (10.4)	27.1 (10.5)	26.8 (9.8)	25.8 (9.4)	25.7 (8.2)
% Male	68	68.5	66.2	66.9	63.7
Mean (SD) baseline VA (LogMAR letters)	63.4 (20.0)	64.7 (19.5)	66.5 (18.6)	65.8 (19.4)	65.2 (18.3)
Mean (SD) baseline Kmax (D)	56.6 (8.2)	56.0 (8.1)	55.7 (7.8)	55.0 (7.5)	55.3 (7.1)
Mean (SD) baseline K2 (D)	50.3 (6.0)	50.0 (5.8)	50.2 (6.2)	49.8 (5.5)	49.7 (6.2)
Mean (SD) baseline TCT (μm)	458.7 (42.1)	462.1 (43.6)	463.6 (44.3)	468.1 (47.7)	469.1 (45.6)

*Note*: Mean values are given with standard deviations in brackets.

Abbreviations: Kmax, maximum keratometry (dioptres); K2, steep keratometry (dioptres); TCT, thinnest corneal thickness (μm); VA, visual acuity (logMar letters).

### 3.2 Treatment protocol trends

The most commonly utilised epithelium‐off CXL UV protocols were the traditional Dresden and accelerated protocols. Of those treatments with 1‐year follow up, the Dresden protocol (30‐min UV exposure) was used in 314 treatments (31.8%) and accelerated (10‐min UV exposure) in 569 treatments (57.7%). Other common protocols included 9‐min UV duration in 37 (3.8%), 8‐minute duration in 32 (3.2%) and 4‐minute duration in 19 treatments (1.9%). Most of the CXL procedures (926, 93.9%) were performed with continuous (no‐pulse) UVA light. Similarly, a majority of CXL procedures (57.8%) were performed with alcohol application followed by manual (19.3%) method. Bandage contact lenses were used post‐operatively in 69.9% of eyes. A majority of the CXL procedures (*n* = 579, 58.7%) were performed with isotonic riboflavin. Riboflavin soaking was most frequently applied for 30 min (74.7% of eyes) with other common protocols including 15 min (10.4%) and 10 min (6.4%).

### 3.3 Cross‐linking effects over time

#### 3.3.1 Visual acuity

VA improved gradually over the 5‐years of follow‐up. The percentage of patients who used the same optical correction methods at baseline and follow‐up was 74.5% in year 1, 73.6% in year 2, 70.7% in year 3, 69.1% in year 4, and 73.5% in year 5. There was a significant improvement in mean VA from baseline by 3.7 logMAR letters (95% CI: 1.8, 5.7; *p* < 0.001) in year 1, 4.9 (95% CI: 2.6, 7.2; *p* < 0.001) in year 2, 4.6 (95% CI: 2.0, 7.3; *p* < 0.001) in year 3, 6.3 (95% CI: 3.1, 9.6; *p* < 0.001) in year 4 and 6.9 (95% CI: 3.1, 10.6; *p* < 0.001) in year 5.

#### 3.3.2 Kmax

Kmax exhibited significant mean flattening of 1.2 dioptres in year 1 (*p* < 0.01). During subsequent years this Kmax flattening appeared sustained but this was not significant. Mean change in Kmax from baseline was found to be −1.2 dioptres (95% CI: −2.0, −0.5; *p* < 0.01) in year 1, −1.2 dioptres (95% CI: −2.2, −0.1; *p* = 0.14) in year 2, −1.1 dioptres (95% CI: −2.3, 0.0; *p* = 0.18) in year 3, −0.8 dioptres (95% CI: −2.3, 0.6; *p* = 0.27) in year 4, and − 1.3 dioptres (95% CI: −2.9, 0.4; *p* = 0.26) in year 5.

#### 3.3.3 K2

K2 flattened significantly from baseline in year 1, after which flattening was not significant. Mean change in K2 from baseline was −0.8 dioptres (95% CI: −1.3, −0.2; *p* = 0.02) in year 1, −0.9 dioptres (95% CI: −1.7, −0.2; *p* = 0.06) at year 2, −1.0 dioptres (95% CI: −1.9, −0.1; *p* = 0.09) in year 3, −0.7 dioptres (95% CI: −1.7, 0.4; *p* = 0.4) in year 4, and − 0.8 dioptres (95% CI: −2.0, 0.5; *p* = 0.4) in year 5.

#### 3.3.4 Thinnest pachymetry

**FIGURE 1:**
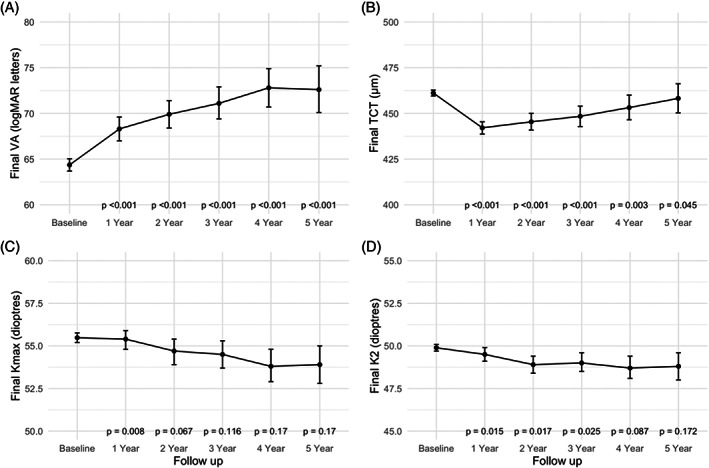
Line plots of final measurements from keratoconic eyes post cross‐linking with 95% confidence intervals at 1–5 years follow‐up. *p*‐Values assessing the change from baseline were evaluated using paired t‐tests. (A) VA, visual acuity (logMAR letters), (B) TCT, thinnest corneal thickness (μm), (C) Kmax, maximum keratometry (dioptres), and (D) K2, steep keratometry (dioptres)

TCT was significantly thinner than baseline which was maintained until year 5. Mean TCT change from baseline was ‐17 μm (95% CI: −21, −12; *p* < 0.001) in year 1, −17 μm (95% CI: −23, −11; *p* < 0.001) in year 2, −15 μm (95% CI: −23, −8; *p* < 0.001) in year 3, −15 μm (95% CI: −23, −8; *p* = 0.002) in year 4, and ‐11 μm (95% CI: −22, 0; *p* = 0.044) in year 5. These results are summarised in Table 2 and Figure 1.

**TABLE 2:** Baseline and final characteristics of keratoconic eyes with 1–5 years follow‐up post cross‐linking, inclusively

Outcome	Year	Baseline (95% CI)	Final (95% CI)	*p*‐Value
Visual acuity	1	**64.6 (63.2, 65.9)**	**68.3 (67, 69.6)**	**<0.001**
	2	**64.7 (63, 66.4)**	**69.9 (68.4, 71.4)**	**<0.001**
	3	**66.5 (64.6, 68.4)**	**71.1 (69.4, 72.9)**	**<0.001**
	4	**65.8 (63.4, 68.3)**	**72.8 (70.7, 74.9)**	**<0.001**
	5	**65.2 (62.4, 68)**	**72.6 (70.1, 75.2)**	**<0.001**
Kmax	1	**56.6 (56.1, 57.2)**	**55.4 (54.8, 55.9)**	**<0.01**
	2	56 (55.2, 56.7)	54.7 (53.9, 55.4)	0.07
	3	55.7 (54.9, 56.6)	54.5 (53.7, 55.3)	0.12
	4	55 (54, 56)	53.8 (52.9, 54.8)	0.17
	5	55.3 (54.2, 56.4)	53.9 (52.8, 55)	0.17
	1	**50.3 (49.9, 50.7)**	**49.5 (49.1, 49.9)**	**0.02**
	2	**50 (49.5, 50.5)**	**48.9 (48.4, 49.4)**	**0.02**
K2	3	**50.2 (49.5, 50.9)**	**49 (48.5, 49.6)**	**0.03**
	4	49.8 (49, 50.5)	48.7 (48.1, 49.4)	0.09
	5	49.7 (48.7, 50.7)	48.8 (48, 49.6)	0.17
	1	**458.7 (455.9, 461.5)**	**442.1 (438.7, 445.4)**	**<0.001**
	2	**462.1 (458.2, 466)**	**445.4 (440.9, 450)**	**<0.001**
TCT	3	**463.6 (458.9, 468.4)**	**448.4 (442.8, 454)**	**<0.001**
	4	**468.1 (461.8, 474.3)**	**453.2 (446.5, 460)**	**<0.01**
	5	**469.1 (462, 476.2)**	**458.2 (450.3, 466.2)**	**0.045**

*Note*: Mean values are given with 95% confidence intervals in brackets. Statistical significance of change from baseline were evaluated using paired *t*‐tests with significant results in bold.

Abbreviations: Kmax, maximum keratometry (dioptres); K2, steep keratometry (dioptres); TCT, thinnest corneal thickness (μm); VA, visual acuity (logMAR letters).

### 3.4 Good and poor responders

#### 3.4.1 Visual acuity

**FIGURE 2:**
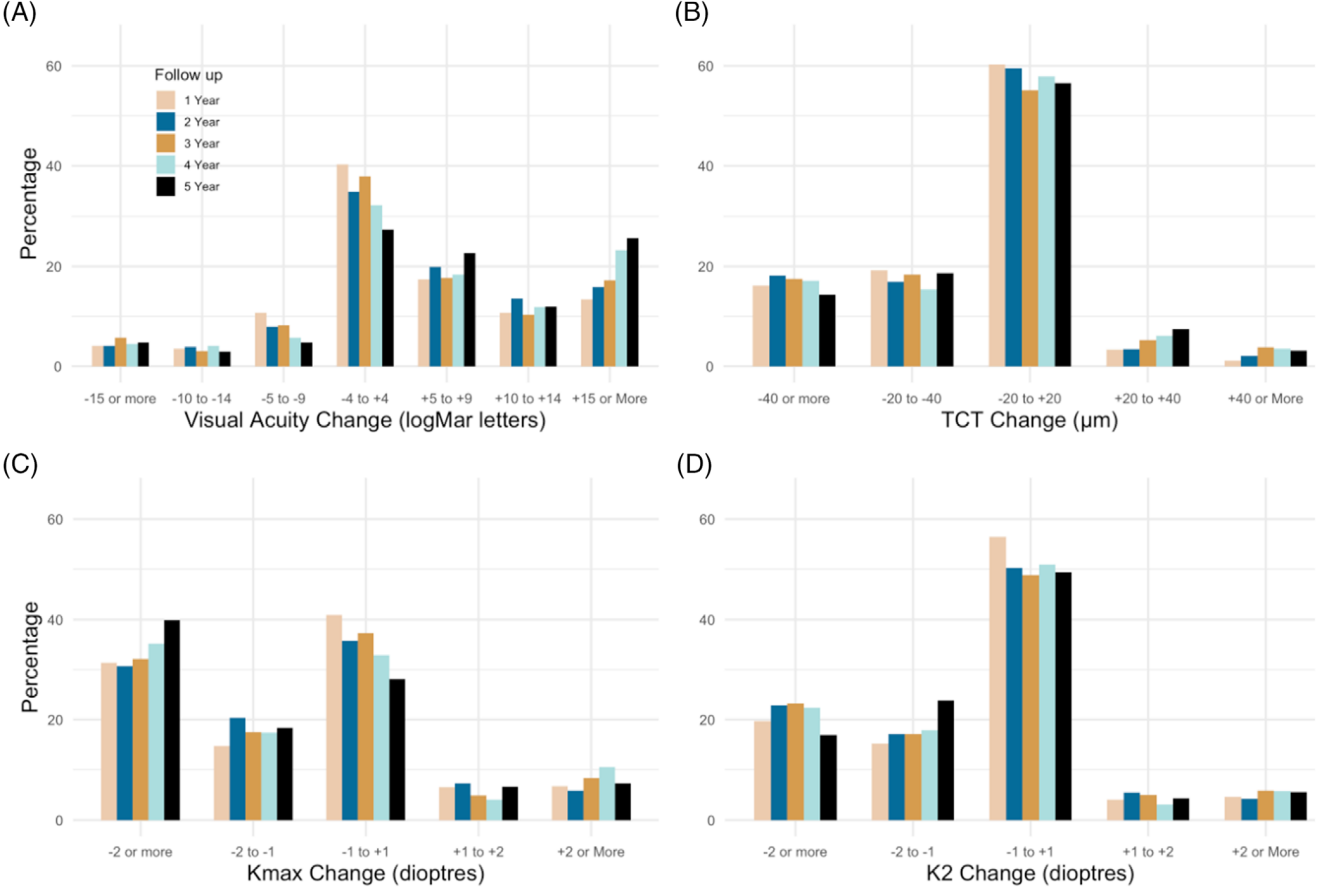
Bar plots of change in clinical parameters from keratoconic eyes post cross‐linking for good and poor responder subgroups at 1–5‐years follow‐up. *p*‐Values assessing the change from baseline were evaluated using paired *t*‐tests. (A) VA, visual acuity (logMar letters), (B) TCT, thinnest corneal thickness (μm), (C) Kmax, maximum keratometry (dioptres), and (D) K2, steep keratometry (dioptres)

The most common outcome was stability in vision (−4 to +4 logMAR letter change) with 40.1% (295/975) of eyes displaying this at 1‐year and 28.4% (46/168) at 5‐years. Over the 5‐year follow‐up, there was a reduction in the proportion of stable eyes and those losing 5–9 letters, and an increase in eyes gaining 5–9 letters and 15 or more letters. The proportion of eyes gaining 5–9 letters increased from 17.5% (129/975) at 1‐year to 22.2% (36/168) at 5‐years. There was an increase in eyes gaining 15 or more letters from 13.4% (99/975) at 1‐year to 24.7% (40/168) at 5‐years. Eyes losing 5 to 9 letters comprised 10.7% (79/975) of 1‐year completers and 4.3% (7/168) of 5‐year completers. At 1 year, 4.1% (30/975) of patients were poor responders to CXL in terms of VA, losing 15 letters (3 logMAR lines) or more. Most patients (83.3%, 25/975) who lost 3 lines or more of VA at 1 year had an adverse event; most commonly haze, scarring, or persistent epithelial defect. Three‐line losers had associated tomographical changes; Kmax steepened 0.8D (range −3.5 to 7.3, SD 3.1); K2 steepened 1.0D (range −2.3 to 11.4, SD 2.9) and TCT thinned 25 microns (range −114 to –11, SD 29). This indicated that some patients in this group significantly progressed or flattened (more than 2 SD). Of the 3‐line losers, 70% (21/975) had better pinhole visual acuity than their habitual acuity. Uncorrected refractive error and/or irregular astigmatism could account for some of this reduction in vision loss. The proportion of 1‐year poor responders losing 3‐lines remained similar at 4.9% (8/168) at 5‐years. Full details across follow‐up years are shown in Figure 2.

#### 3.4.2 Kmax

The most common outcomes were stability in Kmax (+1 to −1 dioptre change)^31^ and 2 or more dioptres of flattening. Over the follow‐up there was a reduction in the proportion of stable patients: 41.0% (347/975) at 1‐year to 28.6% (42/168) at 5‐years and an increase in those flattening by 2 or more dioptres from 31.2% (264/975) at 1‐year to 38.1% (56/168) at 5‐years. The proportion of poor responders to CXL in terms of Kmax remained constant: 5.9% (26/975) steepening by 2 dioptres or more at 1‐year and 7.5% (11/975) at 5‐years (Figure 2).

#### 3.4.3 K2

The majority of patients had K2 readings which remained stable (−1 to +1 dioptre change) over the follow‐up. The proportion of stable patients remained largely unchanged from 56.8% (500/975) at 1‐year to 50.6% (78/168) at 5‐years. The proportion of eyes flattening by 1–2 diopters increased from 15.0% (132/975) of 1‐year to 24.7% (38/168) of 5‐year completers. The proportion of K2 poor responders remained fairly stable with 4.7% (41/975) steepening by 2 dioptres or more at 1‐year and 5.8% (9/168) at 5‐years (Figure 2).

#### 3.4.4 Thinnest corneal pachymetry

Most patients maintained a stable TCT (−20 μm to +20 μm change) over follow‐up, 60.4% (532/975) at year‐1 and 55.5% (86/168) at year‐5. There was a small increase in the proportion of eyes with 20–40 μm thickening from 3.1% (27/975) of 1‐year to 7.7% (12/168) of 5‐year completers (Figure 2).

#### 3.4.5 Adverse events

The most frequently occurring adverse events across 1–5‐year completers were clinically significant corneal haze and corneal scarring. Corneal haze was reported in 147 (15.1%) in year 1, 14 (2.8%) in year 2, 4 (1.1%) in year 3, 4 (1.7%) in year 4, and 3 (1.9%) eyes in year 5. Scarring was recorded in 28 (3.0%) in year 1, 2 (0.4%) in year 2, 2 (0.6%) in year 3, 2 (0.5%) in year 3, and 1 (0.6%) eye in year 5. Other adverse events were infrequent and mostly reported in year 1 (Table 3). No eyes experienced herpetic keratitis, corneal melt, infectious or non‐infectious endophthalmitis, or macula oedema.

**TABLE 3:** Number and percentage of keratoconic eyes post cross‐linking with complete follow‐up with adverse events reported in years 1–5

Adverse event	Year 1 Eyes	%	Year 2 Eyes	%	Year 3 Eyes	%	Year 4 Eyes	%	Year 5 Eyes	%
Total eyes	975		517		367		245		168	
Haze	147.0	15.1	15.0	2.9	4.0	1.1	4.0	1.6	3.0	1.8
Scarring	28.0	2.9	2.0	0.4	2.0	0.5	2.0	0.8	2.0	1.2
Microbial keratitis	6.0	0.6	0.0	0.0	0.0	0.0	0.0	0.0	0.0	0.0
Persistent epithelial defect	15.0	1.5	0.0	0.0	0.0	0.0	1.0	0.4	1.0	0.6
Recurrent erosion	5.0	0.5	3.0	0.6	2.0	0.5	0.0	0.0	1.0	0.6
Sterile infiltrates	3.0	0.3	1.0	0.2	0.0	0.0	1.0	0.4	0.0	0.0
Steroid response	3.0	0.3	0.0	0.0	0.0	0.0	0.0	0.0	0.0	0.0

## 4 DISCUSSION

We report the largest long‐term study of CXL to date, with 976 eyes at 1‐year and 162 eyes at 5‐years. Our data shows that the routine clinical practice outcomes of CXL across Australia, New Zealand, Italy, France, and Spain are similar to those of major RCTs. Our main findings were that overall VA after CXL improves steadily over 5‐years and that Kmax flattens significantly at 1‐year and is then sustained, but not statistically significant in subsequent years. Some patients responded poorly to CXL with 4.1%–7.5% of eyes failing to stabilise, not only by 1‐year, but also at 5‐years. Significant adverse events occurring included clinically significant haze, scarring, persistent epithelial defects, and recurrent erosions. These adverse events though uncommon are important for clinicians and patients to be aware of as they may result in visual loss and ocular pain and/or discomfort as well as predisposing the patient to infection. The most commonly used CXL protocols included accelerated 10‐min UV exposure, riboflavin soaking for 30 min and the use of a bandage contact lens post‐op.

The VA improvement in our study was similar to that found in RCTs and long‐term case series investigating CXL. The largest RCT of CXL for keratoconus to date was carried out by Hersh et al.^12^ and consisted of 100 cross‐linked eyes and controls, also reported clinical stability with a mean improvement in VA of 5.7 letters at 12 months compared to 3.7 letters in our study. One notable difference in our findings is that our data shows a greater proportion of poor‐responders losing 15 letters or more than Hersh's RCT (4.1% vs. 1.0%). Heterogeneous epithelium‐off CXL protocols and younger mean patient age in our study are possible reasons for this difference. Other case series with 5‐year follow‐up, including Hashemi et al^32^ and O'Brart et al,^7^ and have reported slightly lower improvements in corrected visual acuity: a 6‐letter and 1‐letter improvement in BCVA respectively, but did not report the rate of poor responders. We found 4.9% of eyes lost 3 or more lines at 5 years. This rate of long‐term visual loss is not insignificant and both clinicians and patients should be aware of it.

Our study has corroborated other reports^12^ and long‐term case series^13^ that Kmax remains stable overall after CXL, with Kmax change at 5‐years reported as −0.1 dioptres^32^ to −0.9 dioptres.^7^ However, we identified 7.5% of patients were “poor‐responders” at 5‐years: with steepening by 2 dioptres or more. We were unable to find any published long‐term reports with comparable populations investigating different keratometric responses to CXL. Padmanabhan et al^14^ published a retrospective series of paediatric patients from a single centre in Chennai, India. They reported from 59 eyes at 4–6 years and showed a poor response to CXL with >1D steepening of Kmax in 24%. Our mixed adult and paediatric population had a lower rate than this of 14.3% steepening. Padmanabhan's higher rate of poor‐responders is likely to be due to their paediatric population as children have been found to have more aggressive progression in Kmax.^33^ In contrast, only 19.1% of our patients with 5‐year follow‐up were under 18 years old.

Little long‐term data on the safety of CXL has been published. Persistent epithelial defect was a concerning adverse event affecting 23% of eyes in Hersh et al's RCT.^12^ However, their definition included any case persisting beyond only 1 week which may have resulted in a higher rate. The SSKR recorded this adverse event when it occurred and a much lower rate of 1.4% was found which is reassuring for CXL safety. Caporossi et al^34^ had no cases of persistent haze, epithelial defects or recurrent erosion in their 44 eyes at 4–5 years and O'Brart et al^7^ reported no adverse events in any of their 36 eyes at 7‐years. We identified only a few eyes with adverse events from CXL at 5‐years. It is likely that these were not seen in other long‐term studies due to a lack of statistical power.^7^, ^34^ Adverse events at 5‐years in our cohort were rare: 3 eyes (1.9%) had clinically significant haze, 1 eye (0.6%) corneal scarring, and 1 eye (0.6%) persistent epithelial defect. Overall, we found CXL to be a generally safe procedure. However, rare side‐effects can carry significant morbidity and it is important clinicians are aware of and communicate these potential risks to patients.

Registry studies are optimised for providing data at large scale and from routine clinical practice but come at the expense of uniformity and homogeneity when compared to RCTs.^35^ Important sources of heterogeneity include the treatment and monitoring protocols of the 41 sites contributing data to this study, which were designed at the discretion of these sites. In addition to no specified management protocols, our study is limited by further heterogeneity from unspecified diagnostic and outcome definitions. For example, the standard of care for CXL indication is progressive keratoconus, though we did not specify this in our registry. Furthermore, recent reports have suggested CXL in certain high‐risk patients groups, such as paediatric patients, without traditional documented progression. Furthermore, as this was a study on real‐world practice, we did not strictly define adverse events in our study such as clinically significant haze and persistent epithelial defect, leading to further heterogeneity in our results.^36^, ^37^

Only epithelium‐off CXL procedures were included in this study to align with the most common clinical practice and protocol characteristics varied. These included intra‐operative factors such as riboflavin type used, soaking duration,^38^ UV‐light power and duration,^27^ as well as post‐operative factors such as the use of bandage contact lenses post‐op and topical steroid or antibiotic choice and course duration. Monitoring heterogeneity included keratometry and pachymetry measurement devices, which are known to have limitations in inter‐device comparability.^39^ In addition, there was no specified removal time for patients' refractive contact lenses before follow‐up visits. However, current evidence for the effect of contact lens warpage over time, particularly in keratoconus, is limited^40^ such that there is no consensus on when these should be removed prior to assessment. A further cause of monitoring heterogeneity was measurement of vision and using “habitual visual acuity.” There are likely differences in the technique for assessing patients' acuity contributing to heterogeneity. For example, some practitioners may push patients to read smaller letters than others. Although habitual visual acuity provides different information from best corrected visual acuity, it is more representative of patients' everyday visual function. Further, it is particularly appropriate in keratoconic patients who often accept suboptimal refractive correction methods to mitigate intolerable side‐effects such as headaches and contact lens discomfort.

We prioritised power in this study to increase the confidence in our conclusions and most effectively study adverse events. As part of this aim our study included both paediatric and adult patients and does not compare the outcomes from specific CXL procedure protocols, which, while providing high power does limit applying the results to those specific populations and settings. Nevertheless, our previous study showed that the outcomes in children were comparable to those of adults in the SSKR population.^41^ Similarly, we showed that the outcomes with the Dresden (standard) and accelerated UVA protocols,^42^ and the CXL procedures with standard and accelerated riboflavin induction times^38^ were comparable.

Including both eyes from a patient could be considered another limitation of the study. However, keratoconus is usually asymmetrical affecting one eye more than the other. It is therefore a common practice in keratoconus research to include both eyes in the analysis.^43^, ^44^ Similarly, inter‐patient differences on the use of tomography devices should be noted. However, the patients were likely to have been assessed by the same tomography device at baseline and follow‐up as they visited the same clinical practice. The SSKR collects information on these devices at the practice‐level.

The large‐scale data of this study and being drawn from routine clinical practice are its major strengths. RCTs and single‐site studies employ uniform CXL protocols and RCTs have strict inclusion criteria. These study factors often result in populations and surgical techniques that are not well‐matched to many clinicians' practice which limits the applicability of their findings. In contrast, our study has included routine clinical patients from 41 centres across 5 countries. This real world data will give clinicians confidence when counselling their patients on outcomes of CXL.

In conclusion, we report the current largest long‐term study of CXL from 41 sites across 5 countries, with 976 eyes at 1‐year and 162 eyes at 5‐year follow‐up. Our data from routine clinical practice confirms the stabilising effect of CXL. We found that VA improves steadily over 5‐years and Kmax flattens significantly by 1.2D at 1‐year. We observed 4.1%–7.5% of eyes failed to stabilise from the treatment either early on or in the long term. In addition to early adverse events, 0.6%–3% of eyes experienced clinically significant haze, scarring, or persistent epithelial defect at 5‐years. It is important that clinicians use this information to reassure their patients of the high rate of success of CXL in stabilising their disease long‐term, but also to inform them of the probability of long‐term treatment failure or vision loss. Following CXL long term monitoring of keratoconus is still needed.

## FUNDING INFORMATION

Dr A Ferdi is supported by a Northcote Scholarship. Funding for the Save Sight Keratoconus Registry was provided by Keratoconus Australia, and The Australian Vision Research (formerly known as Ophthalmic Research Institute of Australia, ORIA). Dr H Kandel is the Kornhauser Research Associate.

## CONFLICT OF INTEREST

The author declares that there is no conflict of interest.
